# A Population-based Case–Control Study of Urinary Arsenic Species and Squamous Cell Carcinoma in New Hampshire, USA

**DOI:** 10.1289/ehp.1206178

**Published:** 2013-07-19

**Authors:** Diane Gilbert-Diamond, Zhigang Li, Ann E. Perry, Steven K. Spencer, A. Jay Gandolfi, Margaret R. Karagas

**Affiliations:** 1 Section of Biostatistics and Epidemiology, Department of Community and Family Medicine,; 2 Norris Cotton Cancer Center,; 3 Department of Pathology, and; 4 Department of Surgery, Geisel School of Medicine at Dartmouth, Lebanon, New Hampshire, USA; 5 Department of Pharmacology and Toxicology, University of Arizona, Tucson, Arizona, USA

## Abstract

Background: Chronic high arsenic exposure is associated with squamous cell carcinoma (SCC) of the skin, and inorganic arsenic (iAs) metabolites may play an important role in this association. However, little is known about the carcinogenicity of arsenic at levels commonly observed in the United States.

Objective: We estimated associations between total urinary arsenic and arsenic species and SCC in a U.S. population.

Methods: We conducted a population-based case–control SCC study (470 cases, 447 controls) in a U.S. region with moderate arsenic exposure through private well water and diet. We measured urinary iAs, monomethylarsonic acid (MMA), and dimethylarsinic acid (DMA), and summed these arsenic species (ΣAs). Because seafood contains arsenolipids and arsenosugars that metabolize into DMA through alternate pathways, participants who reported seafood consumption within 2 days before urine collection were excluded from the analyses.

Results: In adjusted logistic regression analyses (323 cases, 319 controls), the SCC odds ratio (OR) was 1.37 for each ln-transformed microgram per liter increase in ln-transformed ΣAs concentration [ln(ΣAs)] (95% CI: 1.04, 1.80). Urinary ln(MMA) and ln(DMA) also were positively associated with SCC (OR = 1.34; 95% CI: 1.04, 1.71 and OR = 1.34; 95% CI: 1.03, 1.74, respectively). A similar trend was observed for ln(iAs) (OR = 1.20; 95% CI: 0.97, 1.49). Percent iAs, MMA, and DMA were not associated with SCC.

Conclusions: These results suggest that arsenic exposure at levels common in the United States relates to SCC and that arsenic metabolism ability does not modify the association.

Citation: Gilbert-Diamond D, Li Z, Perry AE, Spencer SK, Gandolfi AJ, Karagas MR. 2013. A population-based case–control study of urinary arsenic species and squamous cell carcinoma in New Hampshire, USA. Environ Health Perspect 121:1154–1160; http://dx.doi.org/10.1289/ehp.1206178

## Introduction

Nonmelanoma skin cancer is the most common type of malignancy among whites and has considerable related morbidity and health costs (Mudigonda et al. 2010). The incidence rates of both forms of nonmelanoma skin cancer—cutaneous squamous cell carcinoma (SCC) and basal cell carcinoma (BCC)—are dramatically increasing in the United States (Karagas et al. 1999; Rogers et al. 2010). Although BCC is more common, SCC has a greater tendency to metastasize and is responsible for greater mortality (Weinstock et al. 1991). Sunlight exposure, male sex, and older age are well known risk factors for SCC (Preston and Stern 1992).

Chronic exposure to arsenic (As) has been associated with SCC in regions of Taiwan where high exposures through drinking water were common (Tseng et al. 1968; Yeh et al. 1968); however, it is not clear whether lower levels of exposure that are common in the United States also are a risk factor for SCC. We previously reported increased odds of SCC in association with toenail As concentrations > 97th percentile [odds ratio (OR) = 2.07; 95% confidence interval (CI): 0.92, 4.66 compared with toenail As less than the median] in a case–control study in the U.S. state of New Hampshire (Karagas et al. 2001). Furthermore, we found evidence of a positive association with toenail As concentrations > 0.105 μg/g (Karagas et al. 2002), but not with concentrations below that value. Although toenail As concentration is considered a reliable long-term biomarker of As exposure (Garland et al. 1993), toenails primarily accumulate inorganic As (iAs), so our previous analysis could not examine specific As species that result from As metabolism.

Upon ingestion, iAs is methylated into monomethylarsonic acid (MMA^V^), which is then reduced to monomethylarsonous acid (MMA^III^). In a second step, MMA^III^ is methylated to dimethylarsinic acic (DMA^V^), which is then reduced to dimethylarsinous acid (DMA^III^) (Vahter 2002). This process is incomplete, such that iAs, MMA^V^, and DMA^V^ are all present in the urine, the primary route of As excretion (Francesconi et al. 2002). Although MMA^III^ and DMA^III^ have been detected in the urine of people highly exposed to As (Mandal et al. 2001; Valenzuela et al. 2005), they have not been found in most studies of people exposed to lower levels of As (Lindberg et al. 2006; Rivera-Núñez et al. 2011).

Arsenic methylation was initially considered a detoxification process because MMA^V^ and DMA^V^ are considered less toxic than iAs and are easily excreted (Gebel 2002; Moore et al. 1997). However, growing evidence suggests that MMA^III^ and DMA^III^ are more toxic than their pentavalent forms and that they may even be more toxic than iAs (Mass et al. 2001; Petrick et al. 2000; Styblo et al. 2000). Given their differing toxicities, it is likely that various As species have different health effects. Studies of populations exposed to high levels of As suggest that the profile of urinary As species may affect As-related skin pathologies; for example, individuals who have lower concentrations of urinary MMA relative to DMA may be at lower risk of As-induced skin lesions (Kile et al. 2011; Yu et al. 2000), nonmelanoma skin cancers combined (Chen et al. 2003), and BCC (Leonardi et al. 2012). To our knowledge, no studies have specifically examined the association between As metabolites and skin cancer in a U.S. population, nor have any studies focused specifically on SCC. We therefore extended our population-based case–control study in New Hampshire to investigate the relation between individual urinary As species and the incidence of SCC.

## Methods

In collaboration with > 90% of the dermatologists and pathologists practicing in New Hampshire and bordering areas, we identified incident cases of invasive SCC of the skin that were newly diagnosed in residents 25–74 years of age from July 2003 through June 2009. Study staff reviewed pathology log books and medical charts at participating dermatology and pathology clinics in order to identify all eligible SCC cases. We selected a control group of New Hampshire residents from the Center for Medicare and Medicaid services (for those ≥ 65 years of age) and driver’s license records provided by the New Hampshire Department of Transportation (for those < 65 years of age), frequency matched to cases on sex and age (25–35, 36–45, 46–50, 51–59, 60–64, 65–69, and 70–74 years) (Applebaum et al. 2007). To be eligible, cases and controls were required to be English-speaking, mentally competent residents of New Hampshire with a working telephone number. Cases and controls were still eligible to participate if they had a previous SCC that was diagnosed prior to the study period. The enrollment and interviews for cases and controls happened concurrently and at about the same rate over the course of the study. The present analysis includes cases and controls interviewed 1 July 2006–10 August 2011. Of 579 SCC cases confirmed eligible, we enlisted 510 (88%), and of the 594 controls, 483 participated (81%).

After study participants gave written informed consent, a study staff member who was blinded to their case/control status interviewed them in person to obtain sociodemographic, lifestyle, medical, and sun exposure information. Home tap water samples were collected from participants’ homes into a commercially washed (mineral-free) high-density polyethylene bottle that meets U.S. Environmental Protection Agency standards for water collection (I-Chem; Thermo Fisher Scientific, Waltham, MA) by study staff wearing individually packed, clean, powderless gloves and following a strict protocol to limit contamination (Karagas et al. 2001). A urine-collection kit with instructions and materials necessary to collect a first-morning-void urine sample was mailed to the participants. The kit included a prelabeled screw-top, 120 mL collection container containing 30 μL diammonium diethyldithiocarbamate to stabilize As species, an insulated thermos, and instructions to refrigerate the urine sample in the thermos until giving it to the interviewer later that day. In addition, participants were asked to complete a 3-day water, rice, and seafood intake record for the 3 days immediately before urine collection. All procedures and study materials were approved by the Committee for the Protection of Human Subjects at Dartmouth College.

*Water As analysis*. Tap water samples were tested at the Trace Element Analysis Core at Dartmouth using inductively coupled plasma mass spectrometry (ICP-MS) with a quadrupole collision cell 7500c Octopole Reaction System ICP mass spectrometer (Agilent) and helium as a collision gas to remove polyatomic interferences, as described previously by Karagas et al. (2001). The limit of detection (LOD) of these analyses ranged from 0.002 μg/L to 0.075 μg/L, with detectable levels in > 96.7% of the samples. When water As measurements were not available, we set the water As concentration to one-half the LOD. The average coefficient of variation for these analyses was 3.22%.

*Urinary analysis*. Urine samples from cases and controls were frozen at –80°C within 24 hr of collection and shipped on dry ice to the University of Arizona, where they were analyzed using a high-performance liquid chromatography (HPLC) ICP-MS system and previously described methods (Gilbert-Diamond et al. 2011). This As speciation method quantifies the concentration of iAs species (iAs^III^ and iAs^V^) and organic As species (MMA^V^, DMA^V^, and arsenobetaine (AsB). No cases or controls had concentrations below the DMA LOD (0.11 μg/L); 3.6% of cases and 4.5% of controls were below the MMA LOD (0.14 μg/L); 32.3% of cases and 31.8% of controls were below the iAs^III^ LOD (0.15 μg/L); and 82.1% of cases and 87.5% of controls were below the iAs^V^ LOD (0.10 μg/L). Urinary As concentrations < LOD were set to one-half LOD.

We calculated the sum of urinary iAs and methylated As species (ΣAs) by summing concentrations of iAs, MMA, and DMA. AsB was excluded from this analysis because it is thought to pass through the body without being metabolized and is nontoxic (Francesconi et al. 2002). The percentage of each As species (%iAs, %MMA, %DMA) in urine samples was calculated by dividing the concentration of each species by the concentration of ΣAs. We calculated the ratio of urinary MMA to iAs as a biomarker of the primary methylation capability, and the ratio of urinary DMA to MMA as a biomarker of the secondary methylation capability (Chen et al. 2003). Urinary creatinine was measured with a colorimetric assay (Assay #500701; Cayman Chemical, Ann Arbor, MI).

*Data analysis*. We first evaluated the association between individual urinary As species and factors that could potentially influence urinary As concentrations in bivariate regression models among controls (*n* = 447). Urinary As concentrations were natural log (ln)-transformed to improve normality. Self-reported rice consumption, including cooked rice and rice cereals, was converted into equivalents of cooked rice using previously described methods (Gilbert-Diamond et al. 2011). To focus on iAs and its methylation products, we excluded the 275 participants who reported eating seafood in the 2 days before sample collection. We chose a 2-day window based on As excretion dynamics (Buchet et al. 1981), although results were consistent in a sensitivity analysis that used a 3-day window to define nonseafood consumers (data not shown). We then fit individual generalized linear models with a logit transform to estimate ORs for SCC in association with a unit increase in urinary ΣAs, iAs, MMA, DMA, %iAs, %MMA, %DMA, iAs/MMA, and DMA/MMA. To improve normality of urinary As species and reduce the influence of potential outliers, we also fit models with ln-transformed urinary As [ln(As)]. We also estimated ORs for SCC according to tertiles of urinary As concentrations (based on the distribution in the controls) to explore possible nonlinear associations. All models were fit with and without adjustment for sex, age (continuous), body mass index (BMI; continuous), education (high school, college, or graduate school), smoking status (at time of diagnosis/reference age: never, former, current), skin reaction to chronic sun exposure (very tan, moderately tan, mildly tan, or freckle/no tan), and urinary creatinine concentration—a measure of urinary dilution (continuous). Resources were available to measure urinary creatinine only in a subset of samples (*n* = 596), so we used multiple imputation and fully conditional specification methods to account for missing data (van Buuren 2007). Models for As percentages and ratios were also adjusted for water As concentration to control for potential confounding by iAs exposure. We performed a sensitivity analysis in which we restricted analyses to non–rice eaters because rice is known to contain DMA (Williams et al. 2005). To account for DMA introduced by unreported fish consumption, we also did a sensitivity analysis in which we further adjusted for urinary AsB. To explore whether the association between urinary As and SCC varied by duration of current water supply use, we stratified participants according to the median duration of current water supply use (< 17 years or ≥ 17 years) and estimated ORs for SCC using covariate-adjusted logistic regression models with ln-transformed urinary As as the exposure. All regression analyses were performed with SAS, version 9.2 (SAS Institute Inc., Cary, NC).

We used a structural equation model to simultaneously model associations between SCC and urinary As metabolites (iAs, MMA, and DMA), water As concentration, and rice consumption. The three urinary As variables are highly correlated and thus ill-suited to be included in a single logistic regression model. The structural equation model permits iAs, MMA, and DMA to each load differently onto a single latent urinary As variable (UAM) based on their correlation structure; the loading score of each As species indicates its relative contribution to the variable. Robust weighted least-square estimators were used to estimate parameters in the model. We used the comparative fit index (Bentler 1990) and Tucker–Lewis index (Tucker and Lewis 1973) to evaluate the model fit. We calculated the indirect association between water As and SCC and rice consumption and SCC, through the latent UAM variable, and tested its statistical significance using the Sobel *Z* test (Shrout and Bolger 2002). For the purpose of comparison, we also created a UAM model without the assumption of unobserved variables by replacing the latent UAM variable with the simple sum of the three urinary As metabolites in the model. The type I error rate used to define statistical significance is 0.05 for these models. The structural equation model analysis was conducted using Mplus, version 6.12 (Muthén & Muthén, Los Angeles, CA).

## Results

The mean (± SD) time between SCC diagnosis and interview/urine collection was 23 ± 10.2 months. Urine samples were collected from 470 SCC cases (92%) and 447 controls (75%), resulting in a total sample size of 917. The sex and age distributions and the percentage of urban versus rural residence did not differ for eligible cases and those who participated in our study. The sex distribution and percentage of urban versus rural residence also did not differ between identified controls and those who participated in our study; however, controls who did participate tended to be older (63 years vs. 56 years). In cases interviewed for our study, 98.5% of those 25–64 years of age had a valid driver’s license at the time of interview, and 98% of those 65–74 years of age were enrolled in Medicare, thus indicating that most would have been included in the data sources used to identify controls. The sex and age group distributions for SCC cases and controls were similar, and approximately 99% of all participants reported their race as white (Table 1). In addition, urban versus rural residence as well as the type of household water supply and length of use were similar between cases and controls, with 58% of cases and 54% of controls consuming water from a shared or private well and an overall median water supply usage of 17 and 18 years, respectively. The consumption of fish and rice in the 2 days immediately before urine collection was also similar in cases and controls. Compared with controls, cases generally had an increased sun sensitivity, measured as a lower tendency to tan with chronic sun exposure (Table 1). On average, cases also had a lower BMI, higher level of education, and lower likelihood to have ever smoked compared with controls.

**Table 1:** Selected characteristics of squamous cell carcinoma (SCC) cases and controls [*n* (%) or median (IQR)].

Variable	SCC cases (*n *= 470)	Controls (*n *= 447)
Sex
Male	284 (60.4)	258 (57.7)
Female	186 (39.6)	189 (42.3)
Age (years)
< 50	19 (4.0)	34 (7.6)
50–59	91 (19.4)	83 (18.6)
60–69	213 (45.3)	203 (45.4)
≥ 70	147 (31.3)	127 (28.4)
BMI (kg/m^2^)
< 18.5	6 (1.3)	3 (0.7)
18.5–24.9	155 (33.4)	127 (28.6)
25.0–29.9	178 (38.4)	161 (36.3)
> 30.0	125 (26.9)	153 (34.5)
Missing (*n*)	6	3
Race
White	465 (99.8)	439 (98.7)
Nonwhite	1 (0.2)	6 (1.3)
Missing (*n*)	4	2
Skin reaction to chronic sun exposure
Very tan	69 (14.7)	134 (30.0)
Moderately tan	239 (51.1)	235 (52.7)
Mildly tan	121 (25.9)	60 (13.5)
Freckle/no tan	39 (8.3)	17 (3.8)
Missing (*n*)	2	1
Smoking^*a*^
Never-smoker	207 (44.0)	160 (35.8)
Former smoker	207 (44.0)	212 (47.4)
Current smoker	56 (11.9)	75 (16.8)
Highest level of education
High school	116 (24.7)	181 (40.5)
College	192 (40.9)	160 (35.8)
Graduate school	161 (34.3)	106 (23.7)
Missing (*n*)	1	0
Residence
Urban	64 (13.6)	48 (10.7)
Rural	406 (86.4)	399 (89.3)
Household water supply
Public	188 (40.8)	203 (46.4)
Shared well	27 (5.9)	20 (4.6)
Private well or spring	242 (52.5)	214 (48.9)
Other	4 (0.1)	1 (0.2)
Missing (*n*)	9	9
Seafood consumption
Yes	147 (32.5)	128 (30.1)
No	305 (67.5)	297 (69.9)
Missing (*n*)	18	22
Rice consumption
Yes	98 (21.8)	96 (22.6)
No	351 (78.1)	328 (77.4)
Missing (*n*)	21	23
Years used water supply	17 (8–32)	18 (8–34)
Water As (μg/L)	0.33 (0.14–1.11)	0.31 (0.12–0.94)
Urinary As (μg/L)
ΣAs^*b*^	5.27 (3.38–8.52)	4.76 (2.94–8.10)
iAs	0.34 (0.13–0.59)	0.30 (0.13–0.54)
MMA	0.52 (0.31–0.82)	0.45 (0.29–0.75)
DMA	4.37 (2.72–7.25)	3.73 (2.37–6.70)
AsB	7.36 (1.69–29.01)	5.79 (1.13–26.34)
Abbreviations: AsB, arsenobetaine; DMA, dimethylarsinic acid; iAs, inorganic arsenic; IQR, interquartile range; MMA, monomethylarsonic acid. ^***a***^Assessed at time of diagnosis/reference age. ^***b***^Sum of iAs, MMA, and DMA.		

Median urinary ΣAs, iAs, MMA, and DMA concentrations were higher in cases than controls (Table 1). In bivariate analyses restricted to controls, age, BMI, and water As concentration were associated with ln-transformed concentrations of urinary ΣAs, iAs, MMA, and DMA (see Supplemental Material, Table S1). In addition, male sex was associated with higher urinary ln(iAs) and ln(MMA), and a higher education level was associated with higher urinary ln(ΣAs), ln(iAs), and ln(DMA). Seafood consumption was associated with higher urinary ΣAs, largely because of higher urinary DMA; each 4-oz. serving of seafood was associated with an estimated 53% (95% CI: 37%, 71%) increase in urinary DMA concentration.

Among participants who reported not eating seafood in the 2 days prior to urine collection (323 cases, 319 controls), we observed a linear association between SCC and ΣAs, MMA, and DMA (Table 2). After adjustment for sex, age, BMI, education, smoking status, skin reaction to chronic sun exposure, and urinary creatinine the OR of SCC was 1.33 for each 1-μg/L increase in urinary MMA (95% CI: 1.04, 1.70) (Table 2). Urinary ΣAs and DMA were also associated with increased odds of SCC. Positive associations with SCC were also observed after ln-transforming the urinary As variables. The OR for each ln-transformed microgram per liter increase of ln(ΣAs) was 1.37 (95% CI: 1.04, 1.80). The ORs for each 1-ln(μg/L) increase in ln(MMA) and ln(DMA) were 1.34 (95% CI: 1.04, 1.71) and 1.34 (95% CI: 1.03, 1.74), respectively. In contrast, SCC was not associated with the percentage of urinary As species or As methylation ratios (see Supplemental Material, Table S2). Results were not sensitive to creatinine adjustment (data not shown). In addition, results were not substantially different when using a 3-day window to define seafood consumers, nor after further adjustment for urinary AsB (data not shown). Regression coefficients were similar in unadjusted and adjusted models after excluding an additional 65 cases and 59 controls who reported eating rice in the previous 2 days, although the smaller sample size resulted in slightly wider confidence intervals (see Supplemental Material, Table S3).

**Table 2:** Unadjusted and adjusted ORs (95% CIs) of squamous cell carcinoma (SCC) by urinary As concentration units or tertiles among cases and controls who did not report seafood consumption for the 2 days prior to urine sample collection (323 cases, 319 controls).

Predictor	Unadjusted^*a*^	Adjusted^*b*^
Untransformed urinary As (μg/L)
ΣAs	1.03 (1.00, 1.05)	1.03 (1.00, 1.06)
iAs	1.21 (0.96, 1.53)	1.26 (0.98, 1.61)
MMA	1.30 (1.03, 1.64)	1.33 (1.04, 1.70)
DMA	1.03 (1.00, 1.06)	1.03 (1.00, 1.07)
ln-Transformed urinary As (μg/L)
ln(ΣAs)	1.29 (1.04, 1.60)	1.37 (1.04, 1.80)
ln(iAs)	1.19 (0.99, 1.43)	1.20 (0.97, 1.49)
ln(MMA)	1.26 (1.04, 1.53)	1.34 (1.04, 1.71)
ln(DMA)	1.27 (1.03, 1.56)	1.34 (1.03, 1.74)
Urinary As tertiles (μg/L)^*c*^
ΣAs		
Tertile 1 (< 3.36)	1 (reference)	1 (reference)
Tertile 2 (3.36 to < 5.31)	0.93 (0.63, 1.38)	0.94 (0.60, 1.45)
Tertile 3 (≥ 5.31)	1.39 (0.96, 2.03)	1.43 (0.91, 2.27)
iAs
Tertile 1 (< 0.23)	1 (reference)	1 (reference)
Tertile 2 (0.23 to < 0.45)	0.92 (0.63, 1.36)	0.97 (0.63, 1.48)
Tertile 3 (≥ 0.45)	1.30 (0.89, 1.90)	1.27 (0.82, 1.98)
MMA
Tertile 1 (< 0.35)	1 (reference)	1 (reference)
Tertile 2 (0.35 to < 0.59)	1.01 (0.68, 1.51)	1.06 (0.68, 1.65)
Tertile 3 (≥ 0.59)	1.61 (1.10, 2.35)	1.76 (1.09, 2.84)
DMA
Tertile 1 (< 2.61)	1 (reference)	1 (reference)
Tertile 2 (2.61 to < 4.18)	0.93 (0.63, 1.38)	0.92 (0.60, 1.43)
Tertile 3 (≥ 4.18)	1.47 (1.01, 2.13)	1.53 (0.97, 2.41)
ΣAs, sum of inorganic arsenic (iAs), monomethylarsonic acid (MMA), and dimethylarsinic acid (DMA). ^***a***^From general linear models with the logistic link function and case/control status as the outcome. ^***b***^Models adjusted for sex, age (continuous), BMI (continuous), education (high school, college, graduate school), smoking status at diagnosis/reference age (never, former, current), skin reaction to chronic sun exposure (very tan, moderately tan, mildly tan, freckle/no tan), and urinary creatinine concentration (continuous). ^***c***^Tertiles were calculated using the urinary As distribution of the study controls.		

Urinary MMA concentration was significantly associated with odds of SCC when urinary As was examined by tertiles of exposure (Table 2). Participants in tertile 3 of urinary MMA had 76% higher odds of SCC than those in tertile 1 (OR = 1.76, 95% CI: 1.09, 2.84). Participants in tertile 3 of urinary ΣAs, iAs, and DMA also had increased odds of SCC, although none of the ORs reached statistical significance.

In an analysis stratified by the median duration of current water supply usage, ORs for SCC were positively associated with unit increases in ln-transformed urinary As species in both strata (< 17 years and ≥ 17 years) (Table 3). ORs were somewhat larger among those with a shorter duration of current water usage, but the differences in the slopes between the two strata were not statistically significant.

**Table 3:** Adjusted ORs (95% CIs) of squamous cell carcinoma (SCC) by ln‑transformed urinary As concentration among participants who did not report seafood consumption for the 2 days prior to urine sample (323 cases, 319 controls), with data stratified at the sample median of current water supply use duration.

Predictor	Years using current water supply^*a*^		*p-*Value for difference in ORs^*b*^
	< 17 (163 cases, 159 controls)	≥ 17 (160 cases, 160 controls)	
ln(ΣAs) (μg/L)	1.55 (1.04, 2.32)	1.20 (0.81, 1.80)	0.38
ln(iAs) (μg/L)	1.29 (0.95, 1.76)	1.09 (0.79, 1.50)	0.51
ln(MMA) (μg/L)	1.59 (1.10, 2.32)	1.14 (0.80, 1.64)	0.21
ln(DMA) (μg/L)	1.46 (0.99, 2.14)	1.22 (0.83, 1.79)	0.45
ΣAs, sum of inorganic arsenic (iAs), monomethylarsonic acid (MMA), and dimethylarsinic acid (DMA). ^***a***^From general linear models with the logistic link function, ln-transformed urinary As as the predictor, and SCC case/control status as the outcome; models are adjusted for sex, age (continuous), BMI (continuous), education (high school, college, graduate school), smoking status (never, former, current), skin reaction to chronic sun exposure (very tan, moderately tan, mildly tan, freckle/no tan), and urinary creatinine concentration. ^***b***^*t-*Test on the difference of the slopes, assuming unequal variances.			

In a structural equation model adjusted for the same set of covariates, iAs, MMA, and DMA each contributed significantly to the latent UAM variable (loading scores = 0.80, 0.52, and 0.49, respectively) (see Supplemental Material, Figure S1). The comparative fit index (> 0.90) and the Tucker Lewis Index (> 0.90) indicated a reasonable model fit. We found an association between the latent UAM variable and SCC [OR per 1-SD increase = 2.04 (95% CI: 1.35, 3.10)]. Water As concentration was associated with the latent UAM variable [1-SD increase in UAM for each 10-μg/L increase in water As = 0.32 (95% CI: 0.30, 0.34)]; the indirect association between water As concentration and SCC through UAM was also significant [for every 1-μg/L increase in water As, OR = 1.02 (95% CI: 1.01, 1.04)]. The structural equation model that did not assume unobserved variables (see Supplemental Material, Figure S2) also showed a significant, but weaker, association between ΣAs and SCC [for each 1-unit increase of ΣAs, OR = 1.02 (95% CI: 1.00, 1.03)].

## Discussion

In this U.S. population–based case–control study of participants living in a region with detectable As in drinking water, we estimated positive associations between SCC and urinary ΣAs, MMA, and DMA concentrations. The results of our structural equation model analysis suggest that a latent urinary As variable representing urinary iAs, MMA, and DMA is associated with SCC. In contrast, percentages and ratios of the individual metabolites were not associated with SCC in the study population.

In our analysis, we were interested in ingested iAs and the products of its methylation: MMA and DMA. In our population, where water As concentrations are relatively low, dietary sources of iAs can play a more substantial role in overall As exposure (European Food Safety Authority 2009). For example, previous studies have found rice consumption to be a significant predictor of urinary iAs (Cascio et al. 2011; Gilbert-Diamond et al. 2011). In our adult study population, however, the prevalence of rice consumption was only 22% in the 2 days immediately before urine collection and was not significantly associated with urinary iAs (see Supplemental Material, Table S1). Seafood is the main dietary source of organic As compounds, including AsB, arsenolipids, and arsenosugars, and the latter two compounds are metabolized into DMA (Cullen and Reimer 1989; Francesconi 2010). Other studies have reported seafood consumption as a major predictor of urinary DMA in U.S. populations (Navas-Acien et al. 2011; Rivera-Núñez et al. 2012), as we observed in our study population (see Supplemental Material, Table S1). Given the different biotransformation pathways of iAS and organic As compounds that result in DMA, as well as their potential differing toxicity (Cullen and Reimer 1989), we excluded participants who reported seafood consumption for the 2 days prior to urine collection. Although rice consumption was a significant predictor of urinary DMA in our study (see Supplemental Material, Table S1), we chose not to exclude rice consumers from our analysis because we were interested in all potential sources of ingested iAs. However, results of sensitivity analyses that excluded rice consumers were generally consistent with the main analyses (see Supplemental Material, Table S3).

Previous ecological studies in the As endemic region of southwest Taiwan (Tseng et al. 1968; Yeh et al. 1968) support an association between high levels of As exposure through well water and SCC. Consistent with our previous work using toenail As as a biomarker (Karagas et al. 2001), results from the present study of urinary As suggest that the association between As and SCC extends to lower levels of As exposure. To our knowledge, no other studies have examined urinary As species and SCC in populations with similar levels of As exposure for direct comparison. Studies in more highly exposed populations have examined urinary As species and As-induced skin pathologies. For example, the Health Effects of Arsenic Longitudinal Study (HEALS) in Bangladesh reported that total urinary As was associated with the risk of incident skin lesions [including melanosis and keratosis, known precursors to skin cancer (Sober and Burstein 1995; Woolgar and Triantafyllou 2011)] in a cohort of 10,182 adults (Argos et al. 2011). In a cross-sectional study of 76 residents of an As endemic region of Mexico with drinking water As concentrations ≥ 50 μg/L, urinary MMA^III^ was significantly higher among those with As-related skin lesions (*n* = 55) compared with those without (*n* = 21); however, there was no difference in concentrations of MMA^V^, DMA^III^, DMA^V^, iAs^III^, iAs^V^, or the sum of iAs and methylated As species (Valenzuela et al. 2005). The percentage or ratio of urinary As metabolites is thought to reflect individual differences in the ability to metabolize iAs, and there is some evidence that they reflect increased susceptibility to As-induced skin lesions. For example, in a case–control study (*n* = 76 cases, 224 controls) in southwest Taiwan, Chen et al. (2003) reported that a low urinary DMA:MMA ratio was positively associated with skin cancer (BCC or SCC) in participants with high lifetime exposure to As (> 15 mg/L per year). In another smaller Taiwanese case–control study (*n* = 26 cases, 26 controls), Yu et al. (2000) reported that individuals with > 15.5% MMA had significantly higher odds of skin disorders (including BCC, SCC, and hyperkeratosis/hyperpigmentation) than did those with ≤ 15.5% MMA (OR = 5.5). In 594 cases and 1,041 controls in the HEALS study, urinary %MMA and the DMA:MMA ratio were significantly associated with skin lesions (including keratosis and melanosis) (Ahsan et al. 2007); the top quartile of %MMA (median, 20.2%) was associated with increased odds of skin lesions compared with the lowest quartile (median, 7.5%) [OR = 1.57 (95% CI: 1.10, 2.26)], and the top quartile of DMA:MMA (median, 10.9) was associated with reduced odds of skin lesions compared with the lowest quartile (median, 3.1) [OR = 0.64 (95% CI: 0.44, 0.91)]. In another large Bangladeshi case–control study of skin lesions (including SCC as well as keratosis and melanosis; *n* = 859 cases, 868 controls), Kile et al. (2011) reported an OR of 1.56 (95% CI: 1.15, 2.12) associated with each increase in log_10_ percentage of MMA. At the relatively low levels of As exposure in our population in the present study, SCC was associated with the absolute concentrations of MMA and DMA measured in urine but not the relative proportions of MMA, DMA, and iAs. Thus, it is not clear whether differences in the proportion of As metabolites reflect susceptibility to skin cancers at lower levels of exposure.

The observed association between methylated forms of As and SCC may reflect the carcinogenicity of those methylated forms or their chemical predecessors. iAs is known to be carcinogenic, although the biological mechanisms are not yet fully understood (International Agency for Research on Cancer 2004). In experimental systems, iAs exposure leads to increased generation of reactive oxygen species (ROS), which in turn lead to DNA damage and increased oxidative sensitive gene expression (Shi et al. 2004). For example, in a recent *in vitro* study in human immortalized lung epithelial cells and adenocarcinoma cells, Liu et al. (2011) reported that As-induced ROS generation activated protein kinase AKT and the extracellular signal-regulated kinase (ERK1/2) leading to increased downstream expression of vascular endothelial growth factor (VEGF), an important angiogenesis regulator. iAs may be carcinogenic through promotion of mitochondrial activity, a process that could fuel the increased energy demands of quickly replicating cells, as well as lead to increased ROS generation (Lee et al. 2011). Arsenic exposure may also promote carcinogenesis through altering the methylation of oncogenes and tumor suppressor genes (reviewed by Cheng et al. 2012). This altered DNA methylation may result from a depletion of cellular *S*-adenosylmethionine (Coppin et al. 2008; Reichard and Schnekenburger 2007; Zhao et al. 1997), an essential methyl donor, by the As methylation pathway. It is still unclear whether iAs methylation products are equally—or even more—carcinogenic compared with iAs. In an *in vitro* study of human epidermal keratinocytes, Vega et al. (2001) showed that trivalent and pentavalent MMA and DMA affected cellular viability, proliferation, and cytokine excretion to approximately the same extent as their inorganic counterparts. Another study of human keratinocytes showed evidence that MMA^III^ may be even more cytotoxic than iAs^III^ (Styblo et al. 2000). There is also *in vitro* evidence of greater genotoxicity of MMA^III^ (Mass et al. 2001; Schwerdtle et al. 2003) and DMA^III^ (Schwerdtle et al. 2003) compared with iAs^III^ and that the genotoxicity is mediated by ROS (Nesnow et al. 2002). Further work is necessary to clarify the biological mechanisms of As carcinogenicity and to understand the relative carcinogenicity of iAs and its metabolites in the *in vivo* context.

In the present study we were unable to measure urinary As prior to SCC diagnosis. Therefore, we cannot exclude the possibility that cases could have changed some behaviors related to As exposure after their diagnosis and before urine collection. In addition, it is possible that SCC treatment could have resulted in changes in As excretion; however, this is unlikely because the primary treatment for SCC is surgical removal of the carcinoma. Prospective studies are needed to better understand the temporality of the observed association. Prospective studies can also elucidate the latency period of low-dose As exposure and associated SCC, which is currently unknown.

Another limitation of our study is that we have a single urinary As measurement, which may not adequately reflect long-term exposure. In a U.S. study of Native American populations (*n* = 60), Navas-Acien (2009) found that urinary As excretion (including As concentrations and percent of urinary As species) remained fairly constant over a 10-year period. In a study in Bangladesh that measured urinary As in 196 participants over 2 years, Kile et al. (2009) also found that the concentrations of urinary As species were fairly reproducible over time, but the percent of As species varied. Studies from Chile (*n* = 73) (Hopenhayn-Rich et al. 1996) and the United States (*n* = 81) (Steinmaus et al. 2005) reported that proportions of As species were fairly stable over time, but only over 2 months and 1 year, respectively. Overall, findings from previous studies suggest that a single urinary measurement may be a reliable measure of exposure for a period of months to years. We recognize, however, that the latency period for As exposure could be very long. Our participants tended to use the same water source for many years (median, 17 years); although water As concentration was associated with urinary As (see Supplemental Material, Table S1), other factors such as diet also influence As exposure (European Food Safety Authority 2009). ORs stratified by the duration of current water system use did not significantly differ (Table 3). Our study was also limited by the fact that we were unable to measure As concentrations directly in the tissue of interest, the skin. Further understanding about As metabolism, tissue distribution, and excretion is necessary to understand the biological interpretation of the associations between urinary As and SCC in our study population.

## Conclusion

Using sensitive methods to measure urinary As exposure in a population-based study of SCC in residents of New Hampshire, we observed that urinary concentrations of ΣAs, MMA, and DMA were positively associated with SCC. These findings suggest that common exposure levels may influence cancer risk in the United States and elsewhere.

## Correction

In Table 1 of the manuscript originally published online, the numbers of SCC cases under “highest level of education” were incorrect. In addition, for seafood and rice consumption, the values for cases and controls were switched, and the numbers for “missing” were accidentally included in the “no” totals. The errors have been corrected here.

## Supplemental Material

(1.7 MB) PDF
