# Correction: A Population-based Case–Control Study of Urinary Arsenic Species and Squamous Cell Carcinoma in New Hampshire, USA

**DOI:** 10.1289/ehp.1206178-erratum

**Published:** 2013-07-19

In Table 1 of the manuscript originally published online, the numbers of SCC cases under “highest level of education” were incorrect. In addition, for seafood and rice consumption, the values for cases and controls were switched, and the numbers for “missing” were accidentally included in the “no” totals. The errors have been corrected here.

