# Supplementary material for: A Population-based Case–Control Study of Urinary Arsenic Species and Squamous Cell Carcinoma in New Hampshire, USA
Source: Environ Health Perspect. 2013 Jul 19;121(10):1154–60. doi: 10.1289/ehp.1206178 (PMC3801199; doi:10.1289/ehp.1206178)
Supplement: (1.7 MB) PDF [file ehp.1206178.s001.pdf]

Environmental Health Perspectives doi:10.1289/ehp.1206178

SUPPLEMENTAL MATERIAL

A Population-based Case-Control Study of Urinary Arsenic Species and Squamous Cell Carcinoma in New Hampshire, USA

Diane Gilbert-Diamond[1], Zhigang Li[1], Ann E. Perry[2], Steven K. Spencer[3], A. Jay Gandolfi[4], and Margaret R. Karagas[1]

[1] Section of Biostatistics and Epidemiology, Department of Community and Family Medicine, Geisel School of Medicine at Dartmouth, Lebanon, New Hampshire, USA

[2] Department of Pathology, Geisel School of Medicine at Dartmouth, Lebanon, New Hampshire, USA

[3] Department of Surgery, Geisel School of Medicine at Dartmouth, Lebanon, New Hampshire, USA

[4] Department of Pharmacology & Toxicology, University of Arizona, Tucson, Arizona, USA

Corresponding author: Diane Gilbert-Diamond, Geisel School of Medicine at Dartmouth, Hinman Box 7937, One Medical Center Drive, Lebanon, NH 03756, 603 653 3362 (t), 603 653 9093 (fax), Diane.Gilbert-Diamond@Dartmouth.edu

Supplemental Material, Table S1. Bivariate association between predictors and percent change in urinary arsenic concentrations among controls. (n=447[a])

Predictor	Percent Change in Urinary Arsenic (95% confidence interval) per unit change in predictor [b]			
	sAs[c]	iAs	MMA	DMA
Male	9.6 (-4.7, 25.9)	20.7 (3.3, 41.1)	22.1 (5.8, 41.1)	6.9 (-7.6, 23.9)
Age (y)	-1.0 (-1.8, 0.0)	-1.9 (-2.7, -1.0)	-1.7 (-2.5, -0.9)	-0.9 (-1.7, -0.1)
BMI (kg/m^2)	-1.3 (-2.6, 0.0)	-1.9 (-3.1, -0.5)	-2.1 (-3.2, -0.8)	-1.2 (-2.5, 0.1)
Education Level	12.5 (3.0, 22.9)	13.9 (3.3, 25.6)	6.8 (-2.6, 17.1)	13.2 (3.3, 24.1)
Current vs. Never Smoker	-6.9 (-22.7, 12.2)	5.9 (-13.9, 30.3)	-1.5 (-18.7, 19.5)	-9.0 (-25.0, 10.5)
Former vs. Never Smoker	-5.6 (-17.9, 8.4)	-12.4 (-24.9, 2.3)	-14.7 (-26.1, -1.6)	-4.2 (-17.1, 10.7)
Chronic sun exposure skin reaction	-2.9 (-11.3, 6.5)	0.1 (-9.5, 10.8)	-3.6 (-12.4, 5.9)	-3.0 (-11.8, 6.8)
Water arsenic concentration (µg/L)	2.1 (1.4, 2.9)	2.2 (1.3, 3.0)	2.2 (1.5, 3.0)	2.1 (1.3, 2.9)
Rice consumption (cooked cups/d)	22.5 (1.7, 47.4)	6.7 (-13.3, 31.3)	10.7 (-8.6, 34.0)	26.4 (4.2, 53.3)
Seafood consumption (4 oz. serving/d)	44.1 (29.2, 60.8)	-5.2 (-16.5, 7.7)	-0.8 (-11.8, 11.6)	53.3 (36.9, 71.4)
%Ln(urinary AsB)[d]	0.1 (0.1, 0.2)	0.0 (0.0, 0.0)	0.1 (0.0, 0.1)	0.2 (0.1, 0.2)

[a]Some sample sizes are smaller due to missing values.

[b]From a model with ln-transformed urinary arsenic as outcome and various subject characteristics as predictors. Age, BMI, water As, rice consumption, seafood consumption, and % Ln(urinary AsB) were modeled as continuous predictors; male, current vs. never smoker, and former vs. never smoker were modeled as dichotomous predictors; and categories of education (high school, college, graduate school) and skin reaction to chronic sun exposure (very tan, moderately tan, mildly tan, freckle/no tan) were modeled as ordinal predictors.

[c]Sum arsenic (sAs) is the sum of inorganic arsenic (iAs), monomethylarsonic acid (MMA), and dimethylarsinic Acid (DMA); arsenobetaine was not included in this total.

[d]Marker of seafood consumption

Supplemental Material, Table S2. Unadjusted and adjusted odds ratios (95% confidence intervals) of squamous cell carcinoma by % arsenic species and methylation indices among cases and controls who reported no seafood consumption for the 2 days prior to urine sample (323 cases, 319 controls)

Predictor	Unadjusted[a]	Adjusted[b]
% iAs	1.00 (0.98, 1.04)	1.00 (0.97, 1.04)
% MMA	1.01 (0.97, 1.04)	1.01 (0.97, 1.05)
% DMA	0.99 (0.97, 1.02)	0.99 (0.97, 1.02)
MMA/iAs	1.06 (0.95, 1.20)	1.08 (0.95, 1.23)
DMA/MMA	1.00 (0.97, 1.02)	1.00 (0.97, 1.03)

[a]From general linear models with the logistic link function, the continuous urinary arsenic concentration variable as the predictor, and squamous cell carcinoma case/control status as the outcome.

[b]Models are adjusted for continuous age, BMI, water As concentration, and creatinine concentration, as well as sex, education (high school, college, graduate school), and smoking status (never, former, current), and skin reaction to chronic sun exposure (very tan, moderately tan, mildly tan, freckle/no tan).

Supplemental Material, Table S3. Sensitivity Analysis: unadjusted and adjusted odds ratios (95% confidence intervals) of squamous cell carcinoma by urinary arsenic concentration among cases and controls who reported no rice or seafood consumption for the 2 days prior to urine sample (258 cases, 260 controls)

Variable	Unadjusted[a]	Adjusted[b]
sAs[c] (μg/L)	1.04 (1.01, 1.08)	1.05 (1.01, 1.09)
iAs (μg/L)	1.28 (0.96, 1.71)	1.35 (0.98, 1.86)
MMA (μg/L)	1.41 (1.04, 1.91)	1.51 (1.04, 2.19)
DMA (μg/L)	1.05 (1.01, 1.10)	1.06 (1.01, 1.11)
Ln(tAs[c] (μg/L))	1.27 (1.00, 1.61)	1.33 (0.96, 1.83)
Ln(iAs (μg/L))	1.23 (1.00, 1.51)	1.28 (0.99, 1.65)
Ln(MMA (μg/L))	1.21 (0.97, 1.50)	1.30 (0.96, 1.76)
Ln(DMA (μg/L))	1.25 (0.99, 1.58)	1.29 (0.95, 1.76)

[a]From general linear models with the logistic link function, the continuous urinary arsenic concentration as the predictor, and squamous cell carcinoma case/control status as the outcome.

[b]Models are additionally adjusted for sex, age (continuous), BMI (continuous), education (high school, college, graduate school), and smoking status (never, former, current), and skin reaction to chronic sun exposure (very tan, moderately tan, mildly tan, freckle/no tan), and creatinine concentration.

[c]Sum arsenic (sAs) is the sum of inorganic arsenic (iAs), monomethylarsonic acid (MMA), and dimethylarsinic Acid (DMA); arsenobetaine was not included in this total.

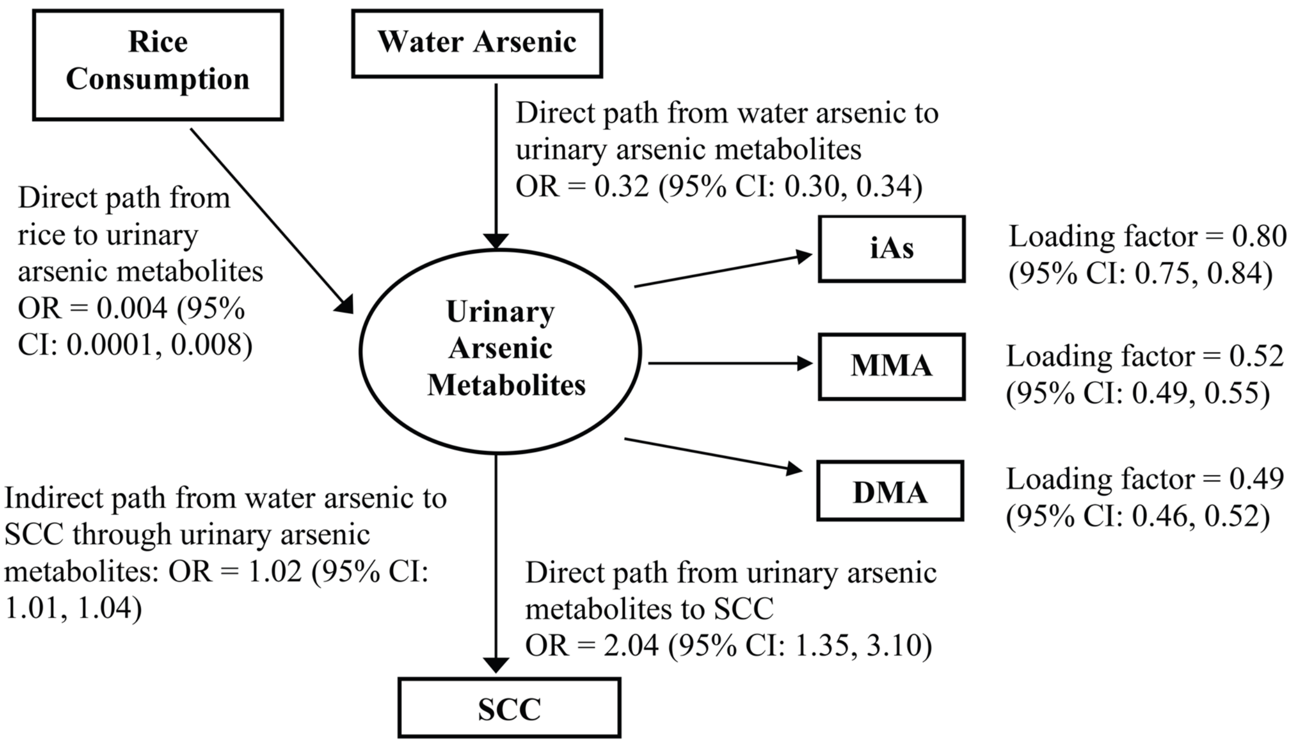

Supplemental Material, Figure S1. Structural equation model relating arsenic exposure from water and rice to odds of squamous cell carcinoma (SCC) through a urinary arsenic metabolites latent variable in subjects who did not consume fish in 2 days prior to urine collection (n=421). Model is adjusted for sex, age (continuous), BMI (continuous), education (high school, college, graduate school), and smoking status (never, former, current), and skin reaction to chronic sun exposure (very tan, moderately tan, mildly tan, freckle/no tan), and urinary creatinine concentration (continuous). iAs – inorganic arsenic, MMA – monomethylarsonic acid, DMA – dimethylarsinic acid.

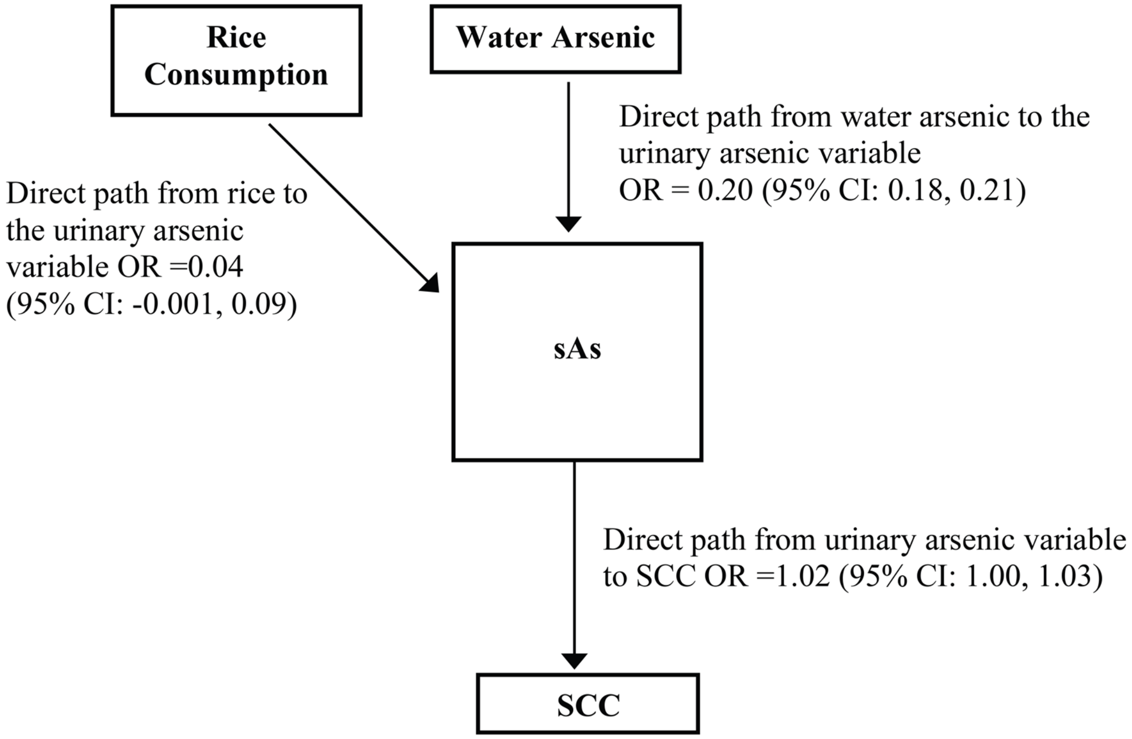

Supplemental Material, Figure S2. Structural equation model relating arsenic exposure from water and rice to odds of squamous cell carcinoma (SCC) through the sum of urinary arsenic concentrations (iAs+MMA+DMA) in subjects who did not consume fish in 2 days prior to urine collection (n=421). Model is adjusted for sex, age (continuous), BMI (continuous), education (high school, college, graduate school), and smoking status (never, former, current), and skin reaction to chronic sun exposure (very tan, moderately tan, mildly tan, freckle/no tan), and urinary creatinine concentration (continuous).
